# Upper Extremity Composite Tissue Allotransplantation Imaging

**Published:** 2013-07-16

**Authors:** Elizabeth George, Dimitrios Mitsouras, Kanako K. Kumamaru, Nehal Shah, Stacy E. Smith, Kurt Schultz, Pamela M. Deaver, Katherine M. Mullen, Michael L. Steigner, Edwin C. Gravereaux, Shadpour Demehri, Ericka M. Bueno, Simon G. Talbot, Bohdan Pomahac, Frank J. Rybicki

**Affiliations:** ^a^Applied Imaging Science Laboratory, Department of Radiology; ^b^Musculoskeletal Radiology, Brigham and Women's Hospital and Harvard Medical School, Boston, Mass; ^c^Toshiba Medical Research Institute, Vernon Hills, Ill; ^d^Division of Vascular Surgery, Department of Surgery; ^e^Division of Plastic Surgery, Department of Surgery, Brigham and Women's Hospital and Harvard Medical School, Boston, Mass

## Abstract

**Objective:** Upper extremity (UE) transplantation is the most commonly performed composite tissue allotransplantation worldwide. However, there is a lack of imaging standards for pre- and posttransplant evaluation. This study highlights the protocols and findings of UE allotransplantation toward standardization and implementation for clinical trials. **Methods:** Multimodality imaging protocols for a unilateral hand transplant candidate and a bilateral mid-forearm level UE transplant recipient include radiography, computed tomography (CT), magnetic resonance (MR) imaging, catheter angiography, and vascular ultrasonography. Pre- and posttransplant findings, including dynamic CT and MR performed for assessment of motor activity of transplanted hands, are assessed, and image quality of vessels and bones on CT and MR evaluated. **Results:** Preoperative imaging demonstrates extensive skeletal deformity and variation in vascular anatomy and vessel patency. Posttransplant images confirm bony union in anatomical alignment and patency of vascular anastomoses. Mild differences in rate of vascular enhancement and extent of vascular networks are noted between the 2 transplanted limbs. Dynamic CT and MR demonstrate a 15° to 30° range of motion at metacarpophalangeal joints and 90° to 110° at proximal interphalangeal joints of both transplanted hands at 8 months posttransplant. Image quality was slightly better for CT than for MR in the first subject, while MR was slightly better in the second subject. **Conclusion:** Advanced vascular and musculoskeletal imaging play an important role in surgical planning and can provide novel posttransplantation data to monitor the success of the procedure. Implementation of more standardized protocols should enable a more comprehensive assessment to evaluate the efficacy in clinical trials.

Reconstructive surgery using autologous tissue for large defects has several limitations including the potential for poor functional and esthetic outcomes, multiple procedures with associated high costs, prolonged rehabilitation, and morbidity due to the use of native tissue.^1^ Vascularized composite tissue allotransplantation has emerged as a successful alternative.

Upper extremity transplantation is the most commonly performed composite tissue allotransplantation; as of May 2013, the International Registry on Hand and Composite Tissue Transplantation reports that 68 upper extremities have been transplanted on 45 patients (22 unilateral and 23 bilateral). Despite this success, there is an unmet need for protocol standardization ranging from the pretransplantation screening to key elements of postoperative care such as immunosuppression and outcome assessment. To our knowledge, no imaging standards for pre- and posttransplant evaluation have been published in the peer-review literature. Although each patient has a unique defect, and requires some degree of customized imaging, standardized protocols for upper extremity transplantation stand to advance basic and translational science.

In addition to inherent human anatomical variations,^2^ prior reconstructive attempts in hand transplantation candidates result in unexpected anatomy, particularly in vascular structures. Considering the risk of muscle fibrosis and transplanted limb contracture associated with perioperative ischemic injury,^3^ preoperative recipient vascular imaging is important for surgical planning to minimize intraoperative ischemia. Posttransplantation imaging assesses the anastomoses for adequate blood flow.

Recipient preoperative bone assessment is critical to donor selection and donor limb preparation. Posttransplantation, bone alignment, union, and hardware complications are evaluated noninvasively. Nerve regeneration and functional outcomes continue to be a main challenge in hand transplantation. Magnetic resonance (MR) neurography with high spatial resolution can, in general, depict peripheral nerve anatomy and pathology, and these methods may be translated to assess nerve regeneration after transplantation. In addition, real-time visualization of motor activity in various musculoskeletal compartments can potentially be used to assess muscle function.

This report describes the imaging protocols for upper extremity transplantation toward developing a standardized protocol that can be implemented into clinical trials.

## METHODS

### Study subjects

Two subjects signed written informed consent approved by our Institutional Human Research Committee, voluntarily enrolled in clinical trial NCT01293214, and are documented in the US Army Medical Research and Materiel Command's Human Research Protection Office. The first subject is a 24-year-old right-hand dominant man undergoing evaluation for face and right hand transplantation. He had sustained extensive burns while riding a motorcycle, which caught fire; he presented with 90° flexion contracture at the right wrist, severe flexion deformity of the thumb, fused interphalangeal joints, and a nearly absent last digit of the right hand. The relatively less deformed left hand was more functional. The second subject is a 66-year-old male bilateral hand transplant recipient, who had undergone bilateral below knee and below elbow amputations for bilateral hand and foot necrosis secondary to disseminated intravascular coagulopathy from urosepsis. He underwent transplantation of bilateral upper extremities at the mid-forearm level.

### Imaging

The imaging protocol for upper extremity transplantation (Appendix 1) used the following modalities for surgical planning: radiography, catheter angiography, vascular ultrasonography, computed tomography (CT), and magnetic resonance imaging (MRI). The postoperative imaging includes radiography, catheter angiography, CT, and MRI.

### Postprocessing for CT and MR

All images were transferred to Vitrea Version 6.2 workstation (Vital Images, Minnetonka, Minnesota) for postprocessing and interpretation. Arteries and veins were identified and segmented using automated vessel selection, as provided by the software, and supplemented by manual correction where necessary. Three-dimensional (3D) volume rendered images were created for assessment of osseous fusion and vessel anatomy. Cine loops were created for MR angiography and dynamic hand movement evaluation.

### Image quality assessment

For both CT (thin-slice axial images) and MRI (maximum intensity projection of the subtraction images), image quality of vessels and bones (on noncontrast CT only) were scored by 2 observers in consensus, using a 4-point scale based on visually estimated sharpness, image noise, and streak or other artifacts, where 4 = excellent, no artifact; 3 = good, mild artifact; 2 = acceptable, moderate artifact present but images still interpretable; and 1 = unevaluable, with severe artifacts.

## RESULTS

All studies were successfully performed; no complications related to contrast administration were reported. Image quality scores are summarized in Table 1; both modalities achieved at least acceptable quality (score of 2). Regarding the quality of each vessel of interest, CT was slightly better than MR in the first subject and MR was slightly better than CT in the second subject.

### Surgical planning

**Figure 1:**
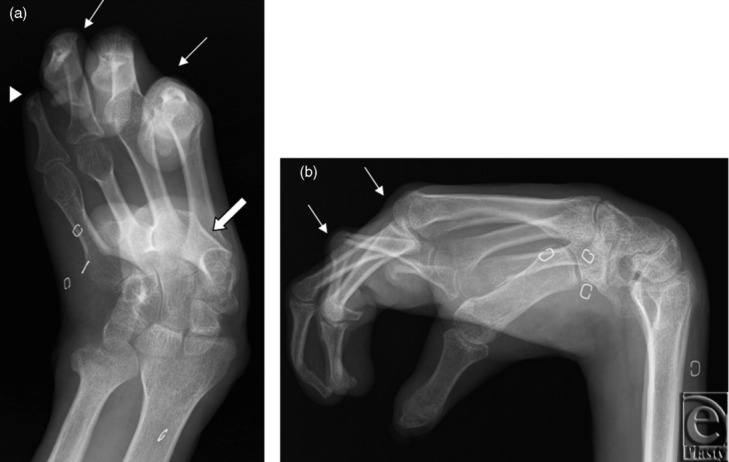
Right hand radiographs. Surgical planning posteroanterior and lateral (*a*, *b*) radiographs of the right hand of subject 1 demonstrate flexion deformity of the wrist and amputation of fifth finger at the proximal interphalangeal (PIP) joint (arrowhead). Flexion deformities of the second and third metacarpophalangeal joints and second through fourth PIP joints are present (arrows). The thumb is held in adduction (block arrow).

**Figure 2:**
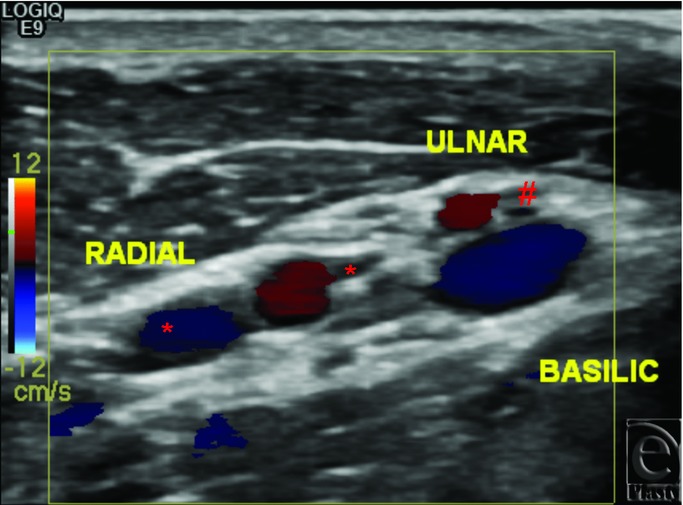
Vascular ultrasonography. Preoperative vascular ultrasonography at the proximal right upper arm of subject 1 demonstrates high arterial bifurcation of brachial into radial and ulnar arteries. Also seen are 2 radial veins (*), one ulnar vein (#), and the basilic vein.

In the first subject, preoperative radiographs of the right hand demonstrated flexion deformity of the wrist, amputation of the fifth finger at the proximal interphalangeal (PIP) joint, and extensive flexion deformities of the metacarpophalangeal (MCP) and PIP joints (Fig 1). Left hand radiographs demonstrated a relatively less deformed extremity with flexion deformity of the wrist and subluxation of the thumb and fifth finger. Both arterial and venous anatomies were complicated from prior injury and reconstructive attempts. An arteriovenous shunt was suggested by the fast circulation time on both CT and MR images; large veins enhanced within 2 to 3 seconds after arterial opacification. There were 3 arteries in the right upper arm, 2 of which were running almost parallel, possibly early branching radial and ulnar arteries. The third artery curved around the humerus and was most likely the deep brachial artery. In the forearm, at least 3 arteries were observed, most likely the ulnar, radial, and interosseous arteries. Although the basilic vein was well-visualized, the cephalic vein was not identified. Large veins joining the basilic vein, plus collaterals, were observed in the forearm. The findings of CT and MRI were confirmed on vascular ultrasonography (Fig 2), which in addition demonstrated no significant arterial disease or venous thrombosis.

**Figure 3:**
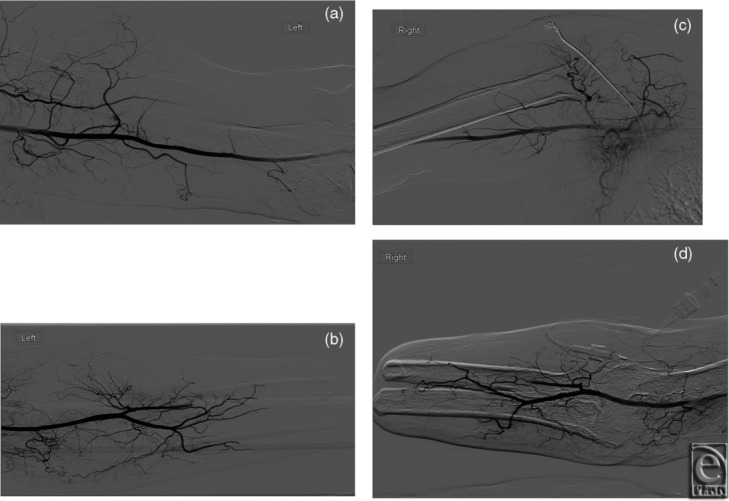
Catheter angiography. Preoperative catheter angiography in subject 2 demonstrates normal vascular anatomy with patent brachial, radial, and ulnar arteries on both left (*a*, *b*) and right (*c*, *d*) sides.

In the second subject, preoperative elbow radiographs demonstrated amputation of the bilateral forearm with hypertrophic changes of the radius and ulnar diaphyses at the stump with no soft tissue abnormality. Catheter angiography demonstrated normal vasculature of bilateral upper extremities with patent axillary, brachial, radial, and ulnar arteries (Fig 3). Preoperative venous mapping by ultrasonography demonstrated patent bilateral subclavian, axillary, cephalic, and basilic veins.

### Postoperative imaging

**Figure 4:**
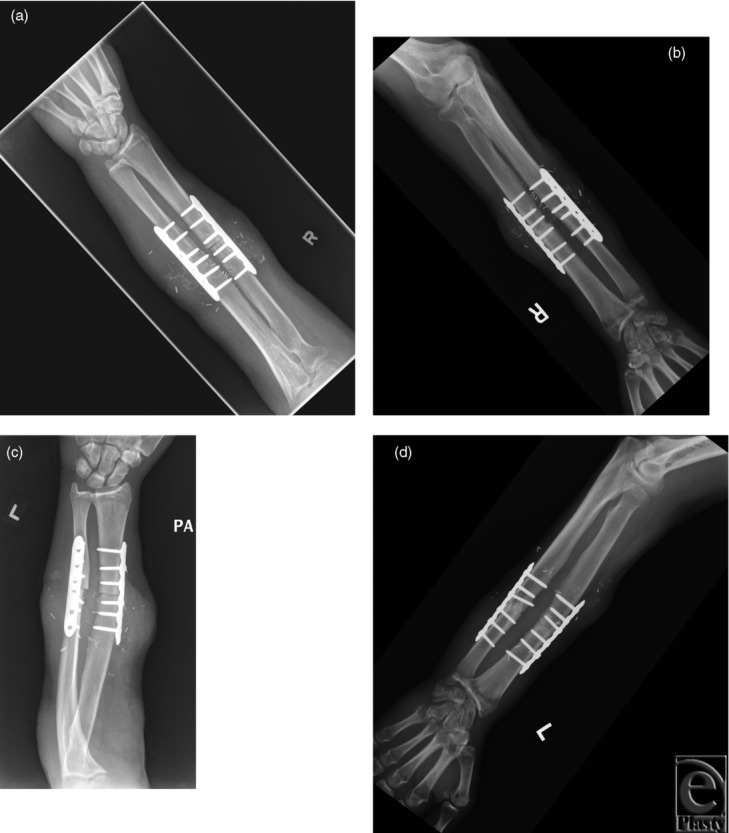
Bilateral elbow radiographs. Frontal radiographs of the bilateral forearm of subject 2 at 1 (*a*, *c*) and 9 months (*b*, *d*) following transplantation demonstrates maturing callus formation and osseous bridging of the radius and ulna at the transplant site. There is interval improvement in soft tissue swelling. No hardware complication is present. Alignment at the elbow and wrist is anatomic.

**Figure 5:**
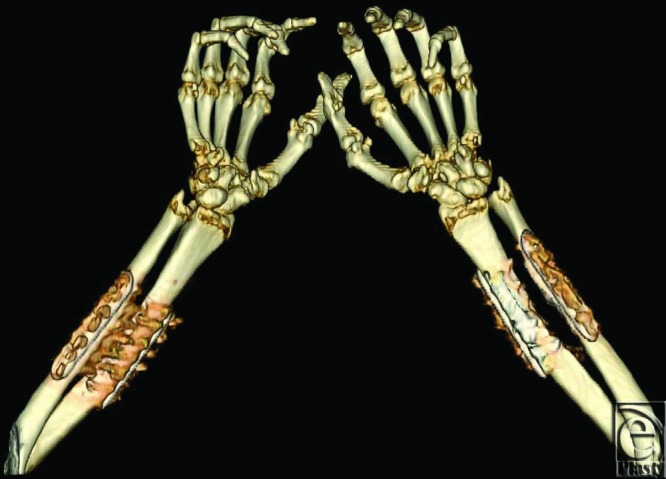
Bilateral upper extremity CT. 3D volume rendered images of both upper extremities obtained 8 months after transplantation demonstrates osseous fusion of the donor and recipient radii and ulnae in subject 2. The fingers are held in flexion at the proximal interphalangeal and distal interphalangeal joints.

In the second subject, immediate postoperative bilateral elbow radiographs demonstrated transplantation at the mid-forearm level with bony fixation using a compression plate and screws. Follow-up imaging excluded hardware complication and demonstrated anatomic alignment with maturing callus formation and osseous bridging (Figs 4 and 5). Dynamic CT and MRI of both transplanted hands demonstrated fingers held in flexion at the MCP, PIP, and distal interphalangeal joints. The range of motion was 15° to 30° at MCP joints and 90° to 110° at PIP joints of both hands (Movies 1 and 2).

Surgical plates implanted in the forearms made vascular interpretation at the anastomotic regions difficult for both CT and MRI. Dynamic MR angiography revealed that both arteries and veins appeared earlier on the left side, and the final vascular networks were more crowded on the left (Movie 3). However, the right hand and forearm had well-opacified arteries and veins, suggesting successful vascular anastomoses, and there was no clinical evidence of ischemia. Catheter angiography also demonstrated patent subclavian, axillary, brachial, ulnar, and radial arteries bilaterally.

## DISCUSSION

With initial success and promising outcomes, upper extremity transplantation is an increasingly available treatment option for numerous individuals with large limb defects from a variety of etiologies.^4^ Although the exact sequence of the transplantation procedure varies, vascular anastomoses and bone fixation have priority in reducing ischemia time and achieving stabilization.^5^ Comprehensive imaging for both surgical planning and surveillance should be guided by standardized protocols. Our group has developed protocols for face transplantation,^6^^-^12 and dynamic vascular imaging has been explored in the setting of lower extremity transplantation.^13^ Imaging to date has relied on wide area detector CT operating in the axial imaging mode^14^^-^16 and advanced MR vascular imaging sequences.^17^^-^21 Upper extremity transplantation requires additional imaging modalities as well as CT and MRI protocols that are tailored to vascular, musculoskeletal, and functional evaluation.

The bony structures, surgical plates, clips, and other implanted material are assessed on radiographs and precontrast CT images. Postcontrast CT and MR images obtained for surgical planning delineate the vascular anatomy for selection of pedicles for anastomoses. The radial and ulnar arteries and the cephalic and basilic veins are the most commonly anastomosed vascular pedicles, with variations depending on the level of limb transplantation. Wide variability in vascular anatomy of the upper limb^2^ and difficulties in localizing recipient vessels in hand transplant patients have both been reported.^22^ Detailed vascular assessment during surgical planning could potentially reduce ischemia time and the risk of perioperative ischemic injury. Catheter angiography and vascular ultrasonography provide supplemental information, especially in cases of incomplete anatomical assessment on CT or MRI due to artifacts.

After transplantation, osseous fusion, alignment, and hardware complications are assessed with radiography and noncontrast CT images. Vascular imaging for surveillance assesses patency of anastomoses and maintenance of vascular supply. When a vascular complication is suspected clinically, for example arterial or venous thrombosis or arteriovenous fistulae,^4^^,^^23^ customized CT and MR protocols fulfill the spatial and temporal resolution requirements.^24^^-^26

When compared to MRI, CT angiography has excellent depiction of small vessels with fewer artifacts among face transplant candidates.^8^ For the subject 1 in this study, vascular image quality on CT was better than MR, mainly due to its higher spatial resolution. However, the asymmetric field of view (FOV) for the upper extremity is much larger in one dimension when compared to the face. Thus, 4-dimensional (4D) CT angiography is challenging, even using a large FOV CT platform.^27^ For subject 2, the MR images had a higher quality score. Second-generation 320-detector row CT scanners,^28^ incorporating radiation dose reduction methods,^29^ will be tested in the near future. Capitalizing MRI on inherent soft tissue contrast has remained most promising to evaluate muscle and tendon movement as well as nerve regeneration.

Aggressive vasculopathy presenting with intimal hyperplasia is a newly recognized indicator of chronic rejection in hand transplantation patients. Current methods to diagnose rejection rely on tissue biopsy, prompted by clinical signs such as transplant rash, erythema, and/or swelling, with the histology being scored using the BANFF scale.^30^^,^^31^ Because this diagnostic algorithm is invasive, time consuming, and there is uncertainty in the interpretation using the BANFF scale, there is a substantial unmet need for noninvasive tools to monitor rejection. Completely restricted to donor vessels, this vasculopathy does not appear to be detectable by standard angiography (CT, MRI, or digital subtraction X-ray) even at a late stage.^32^ Using the clinically based protocols to date, we observed differences in timing of vascular enhancement between right and left transplanted hands; in theory, this could be a sign of silent vasculopathy.

To date, ultrasound biomicroscopy (UBM) (ie, high frequency 2-dimensional [2D] vessel wall ultrasonography) has been reported as a successful tool to measure intimal thickening in hand transplant patients.^32^ However, UBM findings are nonspecific for intimal hyperplasia due to the lack of soft tissue contrast, which only allows monitoring wall thickness. In addition, the 2D nature renders it difficult to perform serial comparative imaging, while penetration depth is limited to a few millimeter at the 55 to 70 MHz used to achieve the necessary resolution,^33^ rendering it only applicable to the most superficial distal arteries (digital, radial/ulnar).

An alternative approach to the assessment of intimal hyperplasia is high-resolution multicontrast 3D MRI^18^^,^^19^; in other vascular beds, longitudinal wall remodeling and thickening due to intimal hyperplasia has been demonstrated.^18^^,^^21^ The rich tissue contrast allows these measurements to be used for testing mechanistic hypotheses regarding the development of intimal hyperplasia.^34^ The “high sampling efficiency” multicontrast MR sequences required^20^ to achieve these resolutions are now widely available in all modern MR systems and may be a useful alternate to UBM in this context. In addition, MR offers other opportunities to explore mechanistic and pathobiology hypotheses regarding vasculopathy, such as direct measure of vessel distensibility.^35^

Functional imaging may also have a more routine role for hand transplantation patients. Return of intrinsic muscle function of the transplanted hand, though variable, has been demonstrated at 9 to 15 months posttransplant, with sustained improvement over more than 5 years.^1^^,^^5^^,^^36^^,^^37^ Advanced CT and MRI allow assessment of dynamic hand movement and range of motion to supplement data obtained from manual measurements and electrophysiology. Current imaging protocols for functional assessment require further optimization to delineate and visualize individual muscle and tendon movement. This could possibly guide the need and sites for tenolyses, which can be required in transplant recipients. Although the effective radiation dose increased about threefold with dynamic imaging, this is less of a concern in extremity imaging, and radiation doses can be lowered using carefully selected protocols for the acquisition and reconstruction of the images.^11^

Magnetic resonance neurography is also a potentially important protocol for hand transplant patients. While we have not completed a study to date, a practical suggested protocol appears in Appendix 2. This method uses diffusion tensor imaging (DTI) to characterize microarchitecture of biologic tissues. In peripheral nerves, it has been shown that DTI detects the integrity of myelin sheaths and fiber bundles. Diffusion tensor imaging–derived fractional normal anisotropy and apparent diffusion coefficient values of the normal median nerve were recently reported, and a statistically significant deviation from those values was found in carpal tunnel syndrome patients.^38^ It would be of interest to similarly correlate changes in fractional normal anisotropy and apparent diffusion coefficient measured longitudinally via MR neurography with clinical assessments in hand transplant function.

In summary, advanced musculoskeletal and vascular imaging is an essential component of evaluation before and after hand transplantation. In addition to standard imaging that can be incorporated to routine protocols, future directions include high-resolution 3D MRI for assessment of chronic rejection and MR neurography for structural and functional evaluation of nerve regeneration.

## Figures and Tables

**Table 1:** Image quality score for the 2 subjects*

**Subject 1**		**Subject 2**		
MR		MR	Right	Left
Radial artery in the upper arm	3	Brachial artery	3	4
Ulnar artery in the upper arm	3	Proximal ulnar artery	4	4
Deep brachial artery	2	Distal ulnar artery	3	4
Radial artery in the forearm	2	Proximal radial artery	4	4
Ulnar artery in the forearm	2	Distal radial artery	4	4
Interosseous artery	2	Palmar arch	3	3
Basilic vein	2	Cephalic vein	3	4
Noncontrast CT		Noncontrast CT		
Humerus	4	Distal radius	4	4
Radius	4	Distal ulna	4	4
Ulna	4	5th Proximal phalanx	4	4
5th Proximal phalanx	3	Arterial phase CT		
Arterial phase CT		Proximal ulnar artery	2	2
Radial artery in the upper arm	4	Distal ulnar artery	3	3
Ulnar artery in the upper arm	4	Proximal radial artery	2	2
Deep brachial artery	3	Distal radial artery	3	3
Radial artery in the forearm	3	Palmar arch	2	2
Ulnar artery in the forearm	3	Venous phase CT		
Interosseous artery	2	Basilic vein	3	4
Venous phase CT				
Basilic vein	3			

*CT indicates computed tomography; MR, magnetic resonance.

**Table 2:** MR neurography protocol*

2D/3D	Sequence type	Sequence name/Plane	TR, ms	TE, ms	Flip angle	Fat suppression	iPAT	Voxel size, mm^3^	Comments
2D	T2	Axial T2	3800	64	150°	SPAIR	Off	0.6 × 0.4 × 3	
2D	T1	Axial T1	913	10	155°	None	Off	0.5 × 0.4 × 3	
3D	T2	Coronal T2 SPACE	1000	96	130°	SPAIR	2	1.0 × 1.0 × 1.0	3D TSE with variable flip angles
3D	Steady-state free precession	Coronal PSIF	10	5	35°	Water excitation	2	0.6 × 0.6 × 2	b-value 80
2D	PD	Sagittal PD	3750	31	150°	SPAIR	Off	0.5 × 0.4 × 3	
3D	STIR	Coronal STIR SPACE	1400	106		None	2	0.8 × 0.8 × 1.0	TI: 220 ms

*iPAT indicates integrated parallel acquisition techniques; PD, proton density; PSIF, reverse fast imaging with steady state precession; SPACE, sample perfection with application optimized contrast using different angle evolutions; SPAIR, spectral adiabatic inversion recovery; STIR, short T1 inversion recovery; TE, echo time; TI, inversion time; TR, repetition time; TSE, turbo spin echo.

**Movie 1:** Left hand MRI. Cine loop of 2D FLASH T1-weighted sequence from MRI of left hand of subject 2 obtained 8 months following transplant demonstrates dynamic range of motion of 15° to 30° at metacarpophalangeal joints and 90° to 110° at proximal interphalangeal joints.

**Movie 2:** Left hand CT. Cine loop reconstructed from dynamic CT demonstrates the range of motion of transplanted hand of subject 2.

**Movie 3:** Bilateral time-resolved MR angiography. Cine loop of maximum intensity projection images demonstrating faster enhancement and denser vascular networks on the left compared to the right transplanted extremity in subject 2.

## Appendix 1. Imaging Protocols

### RADIOGRAPHY

Digital radiography (DRX Evolution, Carestream, Rochester, New York) used a direct capture detector without a grid. Posterior-anterior, oblique, and lateral views of both wrists used standard technique (60 kVp, 3.2 mA, exposure time 12 milliseconds), as did anterior-posterior and lateral views of both forearms.

### COMPUTED TOMOGRAPHY

A 320 × 0.5 mm detector row CT scanner (AquilionONE, Toshiba Medical Systems, Tochigi-ken, Japan) was used for noncontrast multiphase axial imaging; 80 kVp, 300 mA, craniocaudal FOV 16 cm, slice thickness 0.5 mm, gantry rotation time 0.35 second, over 10.5 seconds during dynamic movement (hand folding) of both transplanted hands in subject 2.

Arterial and venous phase static images were acquired helically using the central 64 detectors. For unilateral hand transplant planning in subject 1, the right hand, forearm, and arm were included in FOV: 120 kVp, tube current determined by 3D exposure modulation, slice thickness 1.0 mm with 0.5 mm overlap, and helical pitch of 0.828. For posttransplant imaging of subject 2, bilateral hands and forearm were included in the FOV: 120 kVp, 80 mA, slice thickness 2.0 mm, and helical pitch of 0.641. Contrast enhancement used 75 mL Iopamidol-370 mg iodine per milliliter, (Isovue-370, Bracco Diagnostics, Princeton, New Jersey) injected via a peripheral vein at 3 to 4 mL per second, followed by 40 mL saline, using a power injector (Empower CTS, ACIST Medical, New York, New York). Acquisition timing for the arterial phase was determined by bolus tracking in the proximal aorta with a trigger at 150 Hounsfield Unit; venous phase images used an additional 30 to 40 seconds delay.

The estimated effective radiation dose of 1.06 mSv (subject 1) and 3.39 mSv (subject 2) was calculated from the scanner reported dose length product using a conversion factor *k* = 0.0008 mSv mGy^−1^ cm^−1^.

### MAGNETIC RESONANCE IMAGING

Imaging was performed on a 3-Tesla MR system (Siemens Trio, Erlangen, Germany) using an 8-channel Foot/Ankle coil, 8-channel torso coil, and several spinal coils with integrated parallel imaging technique with acceleration factor of 2 for all sequences. For subject 2, similar to CT imaging, a dynamic continuous scan was performed at first to evaluate the movement of the left hand with a 2D spoiled fast spine echo (2D-FLASH) imaging: TR and TE of 5.7 and 2.46 milliseconds, flip angle 70°, and a pixel size 0.438 × 0.438 mm^2^ with a slice thickness 6 mm.

A 3D-time resolved MR angiography was performed to evaluate the vessels. Eight milliliters of gadolinium contrast (MultiHance, Bracco Diagnostics, Princeton, New Jersey) was injected at a flow rate of 2 mL per second. Image acquisition was manually triggered. The 3D-time resolved MR angiography acquisition was repeated at 5- to 10-second intervals, for a period of 120 to 180 seconds as determined by the patient's extremity circulation time. The scan parameters were slice thickness 1.5 mm, TR and TE of 4.33 to 4.38 milliseconds and 1.43 to 1.46 milliseconds, flip angle 25°, and pixel size 1.027-1.116 × 1.027-1.116 mm.

### CATHETER ANGIOGRAPHY

After common femoral artery access was established with a 5F sheath, aortic arch angiography was obtained with a pigtail catheter. Using a guidewire, an end-hole catheter was advanced into the innominate and subclavian artery to obtain the arm arteriogram in anteroposterior/left anterior oblique view as appropriate, with further advancement into the brachial artery to visualize the distal vasculature in right anterior oblique view. Femoral artery closure was achieved either manually or using 6F Angio-Seal device.

The total duration of conscious sedation was about 90 minutes, with estimated blood loss less than 50 mL. The total fluoroscopy duration was 6.9 to 14.6 minutes with a total radiation dose of 121 to 409.231 mGy. A total of 80 to 150 mL of iodinated contrast (Visipaque 270, GE Healthcare Inc, Princeton, New Jersey) was used.

### VASCULAR ULTRASONOGRAPHY

Duplex ultrasound (Acuson, Siemens, Erlangen, Germany) with color-assisted Doppler examination used linear 5 to 12 MHz transducers on the anatomy of interest to map the vascular tree and assess for the presence of arterial disease or venous thrombosis. In general, vascular measurements were obtained at the proximal, mid, and distal arm and forearm as appropriate/feasible. The arterial velocities were documented to assess for significant atherosclerotic disease. Venous compressibility and Doppler response to respiratory variation and augmentation maneuvers were assessed.

## Appendix 2. MR Neurography

Extremity neurography on a 3-Tesla MR scanner (Siemens Verio, Erlangen, Germany) using a body matrix coil (Table 2). No intravenous contrast utilized.
